# Ultrastable Offset-Locking Continuous Wave Laser to a Frequency Comb with a Compound Control Method for Precision Interferometry

**DOI:** 10.3390/s20051248

**Published:** 2020-02-25

**Authors:** Ruitao Yang, Haisu Lv, Jing Luo, Pengcheng Hu, Hongxing Yang, Haijin Fu, Jiubin Tan

**Affiliations:** 1 Institute of Ultra-Precision Optoelectronic Instrument Engineering, Harbin Institute of Technology, Harbin 150001, China; ruitao.yang@hit.edu.cn (R.Y.); lvhaisu123@stu.hit.edu.cn (H.L.); luojingcetc14@outlook.com (J.L.); takeon@126.com (H.Y.); haijinfu@hit.edu.cn (H.F.); jbtan@hit.edu.cn (J.T.); 2 Key Lab of Ultra-Precision Intelligent Instrumentation, Harbin Institute of Technology, Ministry of Industry and Information Technology, Harbin 150080, China; 3 Postdoctoral Research Station of Optical Engineering, Harbin Institute of Technology, Harbin 150080, China; 4 Nanjing Research Institute of Electronics Technology, Nanjing 210013, China

**Keywords:** frequency locking, optical frequency comb, acousto-optical frequency shifter, compound control

## Abstract

A simple and robust analog feedforward and digital feedback compound control system is presented to lock the frequency of a slave continuous wave (CW) laser to an optical frequency comb. The beat frequency between CW laser and the adjacent comb mode was fed to an acousto-optical frequency shifter (AOFS) to compensate the frequency dithering of the CW laser. A digital feedback loop was achieved to expand the operation bandwidth limitation of the AOFS by over an order of magnitude. The signal-to-noise ratio of the interference signal was optimized using a grating-based spectral filtering detection unit. The complete system achieved an ultrastable offset-locking of the slave CW laser to the frequency comb with a relative stability of ±3.62 × 10^−14^. The Allan deviations of the beat frequency were 8.01 × 10^−16^ and 2.19 × 10^−16^ for a gate time of 10 s and 1000 s, respectively. The findings of this study may further improve laser interferometry by providing a simple and robust method for ultrastable frequency control.

## 1. Introduction

The accuracy of laser interferometry is highly dependent on the stability of the laser wavelength. In some ultra-precision measurement applications (e.g., Laser Interferometer Gravitational-Wave Observatory (LIGO) and semiconductor manufacturing equipment), the laser interferometer works in a vacuum environment [1,2,3]. Without the disturbance from the refractive index of air, the stability of laser frequency becomes the key factor for improving accuracy. 

Owing to the abundant spectral modes with a wide wavelength range and easy traceability to the SI time unit, optical frequency combs revolutionized the fields of precision spectroscopy [4,5], optical time standard [6], frequency metrology [7], and laser ranging [8,9]. However, in some applications, single wavelength continuous wave (CW) lasers are preferred instead of multiple frequency modes with a comb shape [10,11]. To ensure the frequency stability of the CW laser for precise measurement, a locking system is needed with an optical frequency comb as a reference [10,11,12,13,14]. The accuracy and repeatability of the experimental results are hence highly affected by the features of the locking system. 

Conventional locking systems are based on a fast servo loop [12]. Frequency or the phase difference between the CW laser and the adjacent reference comb mode is detected and feedback is controlled via the operation temperature, current, and/or the piezo cavity length regulator of the CW laser [10,11,12,13,14]. In these locking systems, dynamic properties of the control loop should be carefully considered, especially for the response of the laser oscillator and servo actuators. The design and optimization of such servo loops are challenging [12]. Moreover, the realization of these locking systems usually requires fast modulation input ports on the slave laser. However, many commercial CW lasers cannot be precisely controlled with a bandwidth over the kHz level. 

Another promising solution known as the acousto-optic frequency shifter (AOFS)-based feedforward method for laser frequency control was first proposed for direct carrier-envelope phase stabilization of frequency combs [15]. Then, the method was applied to narrow the linewidth of a CW laser referencing to a frequency comb [16,17,18]. Using this method, the coherence properties of the frequency comb can be transferred to the CW laser. However, the long-term locking property of the AOFS-based feedforward method has not been reported. Besides, the limited operation bandwidth of the AOFS is neglected in previous studies. Accordingly, the frequency drift of the slave CW laser cannot be fully compensated.

In this study, we developed a simple and robust system to offset lock a slave CW laser to a reference frequency comb with both digital feedback and analog feedforward compound control. The limitation of the AOFS operation bandwidth was solved by a digital feedback control loop with up to 100 MHz capture range of offset-locking. At the same time, the signal-to-noise ratio (SNR) of the interference signal was improved with a grating-based spectral filtering detection unit by 16 dB, comparing to a direct detection of the interference signal. Consequently, the interference SNR was optimized to only 5.5 dB less than the theoretical upper limit. With the help of large-range digital feedback control and high SNR interference signal detection, the complete system achieved an ultrastable offset-locking of the slave CW laser to the frequency comb. The Allan deviations of the beat frequency were 8.01 × 10^−16^ and 2.19 × 10^−16^ for a gate time of 10 s and 1000 s, respectively. The proposed method may be further applied to precision laser interferometry with ultra-high frequency stability and easy feasibility.

## 2. AOFS Based Analog Feedforward Control Principle and Its Limitation

The basic principle of feedforward control is a real-time correction of the final result based on the direct measurement of disturbance in the process. Therefore, locking a CW laser to a reference frequency comb using the feedforward method first requires the frequency detection of the beat signal between two lasers. Assuming that the frequency of the CW laser *f*_CW_ is less than the frequency of the nearest comb mode *f*_n_, the frequency of the beat signal can be expressed as: *f*_Beat_ = *f*_n_ − *f*_CW_. Feeding the beat signal to an AOFS to up-shift the CW laser frequency, the frequency of the diffraction beam can be compensated back to the frequency of the reference comb accordingly.

**Figure 1:**
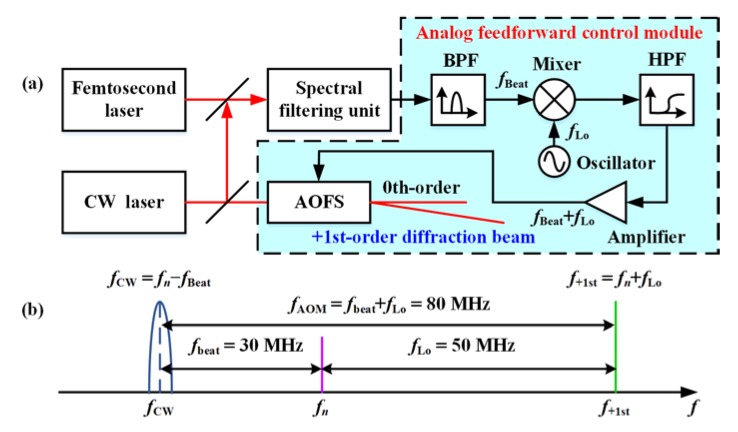
(**a**) The schematic of the analog feedforward control system. (**b**) Spectra of the CW laser, reference frequency comb mode, and the first-order diffraction beam. BPF: Band-pass filter. HPF: High-pass Filter. AOFS: Acoustic-optic frequency shifter. Osc: Oscillator.

In practical applications, the offset frequency between the CW laser and the nearest comb mode often needs to be adjusted. An extra local oscillator of *f*_Lo_ can meet this requirement effectively. In the improved schematic shown in Figure 1a, the beat signal is further mixed with the local oscillator. The high-pass filtering result is served as the AOFS driving signal. In this condition, the frequency of the AOFS driving signal can be expressed as:

(1) $$f_{AOM}=f_{\mathrm{Lo}}+f_{Beat}=f_{\mathrm{Lo}}+f_{n}-f_{\mathrm{CW}}$$

After the first-order diffraction of up-frequency-shifting, the frequency of the CW laser can be expressed as:

(2) $$f_{+1st}=f_{CW}+f_{AOM}=f_{n}+f_{\mathrm{Lo}}$$

The analysis of the signal spectrum is shown in Figure 1b. The beat signal of 30 MHz is mixed with a 50 MHz local oscillator to be the 80 MHz signal for AOFS driving. The linewidth of the first-order diffraction beam is no more limited by the source CW laser. Instead, it has been reduced to the same level of the frequency comb mode. However, the effect of offset-locking will still be limited by the time delay in the feedforward system. Assuming that the system time delay from beat signal detection to the AOFS diffraction is Δ*t*, the spectral density of the first-order diffraction beam frequency variation can be expressed as follows:

(3) $$S_{+1\mathrm{st}}\left(v\right)=S_{n}\left(v\right)+2S_{\mathrm{cw}}\left(v\right)\left(1-\cos2\pi v\Delta t\right),$$

where *v* stands for the offset frequency from the central frequency of the CW laser. Moreover, the corresponding phase noise decreases sharply with increasing frequency *v*. Based on Equation (3), spectral density of the frequency variation will be the same for the first-order diffraction beam and the reference comb mode, when 1–cos2*πv*Δ*t* = 1. The equality requires either the frequency difference *v* or the time delay Δ*t* be zero, which is consistent with our predictions. If the cosine term in Equation (3) can be expanded as a Taylor series, then it can be expressed as follows:

(4) $$\begin{array}{c} 1-\cos2\pi v\Delta t=1-\left(1-\frac{{\left(2\pi v\Delta t\right)}^{2}}{2}+o\left({\left(2\pi v\Delta t\right)}^{2}\right)\right)\\ =2\pi^{2}v^{2}\Delta t^{2}+o\left({\left(2\pi v\Delta t\right)}^{2}\right) \end{array}$$

When the higher-order remainder is neglected, Equation (4) can be simplified to

(5) $$S_{1st}\left(v\right)-S_{n}\left(v\right)={\left(2\pi v\Delta t\right)}^{2}S_{cw}\left(v\right)$$

In this case, the term (2π*v*Δ*t*)^2^ can be regarded as the factor of phase noise suppression. Since the range of offset frequency *v* is limited to the linewidth of the CW laser, which is approximately 100 kHz in our system, the capability of phase noise suppression is mainly determined by time delay Δ*t*. The system time delay Δ*t* is a result of the AOFS module time delay, the photodiode conversion delay, and the electrical signal transfer delay. According to previous studies [16,17,18], the system time delay is typically less than 750 ns. In this case, the factor of phase noise suppression can be over −13 dB for a CW laser with a linewidth of no more than 100 kHz. 

According to the theoretical analysis in [17], the control bandwidth *B* of the AOFS-based feedforward control system can be calculated using the system time delay Δ*t*.

(6) $$B=\frac{1}{6\Delta t}$$

The corresponding control bandwidth of the AOFS-based feedforward control system is above 200 kHz. However, for a conventional feedback control system, such a high control bandwidth usually requires a careful design of the servo units and special FET or bias-T ports for fast frequency modulation.

**Figure 2:**
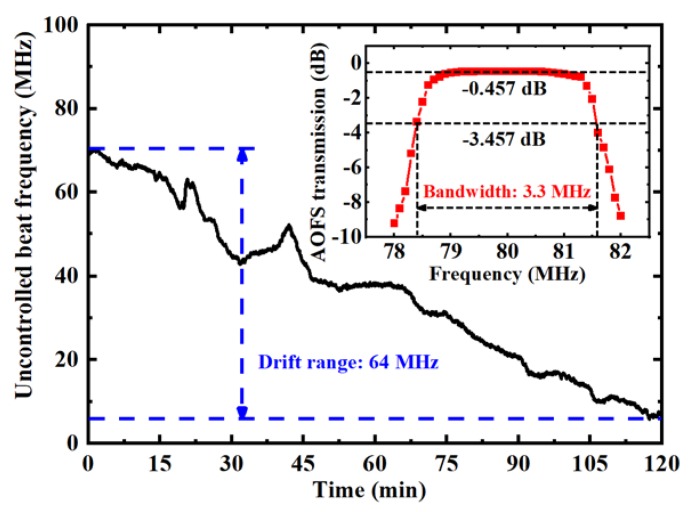
The frequency drift of the beat signal between unlocked CW laser and reference comb mode. The inserted illustration shows the transmission curve and the bandwidth of our AOFS.

The drawback of the AOFS-based feedforward control method for broader applications is its limited locking range. The experimental result of the frequency drift of the beat signal between an uncontrolled CW laser and a reference frequency comb is shown in Figure 2. The frequency drift reaches 64 MHz in 120 min. However, the 3 dB bandwidth of the AOFS is only ~3.3 MHz, as shown in the insertion of Figure 2. Therefore, the AOFS can only compensate the frequency of the CW laser in a frequency range of ±1.65 MHz with the feedforward control. In practical applications, the long-term frequency drift of the CW laser should be reduced below ±1 MHz to keep the AOFS-based feedforward control functional.

## 3. Digital Feedback Control for Extended Frequency Locking Range

**Figure 3:**
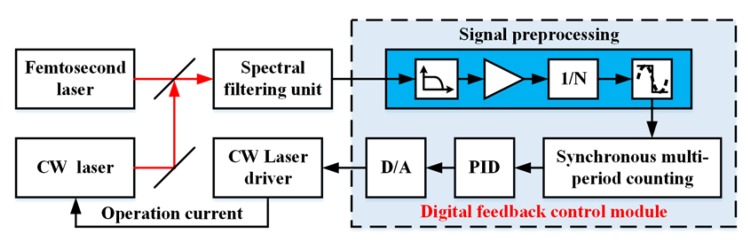
Schematic of the digital feedback control system. The signal preprocessing module is marked with a blue background.

In order to bridge the gap between the frequency drift of the beat signal and the limited bandwidth of the AOFS, a digital feedback control module was designed in the locking system to achieve frequency pre-locking (Figure 3). The detected sinusoidal beat signal was low-pass filtered, amplified, and converted into a digital square wave signal. The low-pass filter was added to suppress the harmonics of comb repetition rate and other beat signals above the frequency of the half comb repetition rate. Thus, the beat signal of the lower frequency between the CW laser and two nearby comb lines was extracted. A multi-period synchronous measurement method was realized in a field programmable gate array to achieve the equal accuracy measurement of the beat signal frequency. Then, we applied a micro-controller to calculate the error between the current and the target frequency of the beat signal. Accordingly, the driving current of the CW diode laser was adjusted with a proportional-integral-derivative (PID) control algorithm. The frequency of the CW laser can then be offset-locked to the reference comb mode coarsely. 

**Figure 4:**
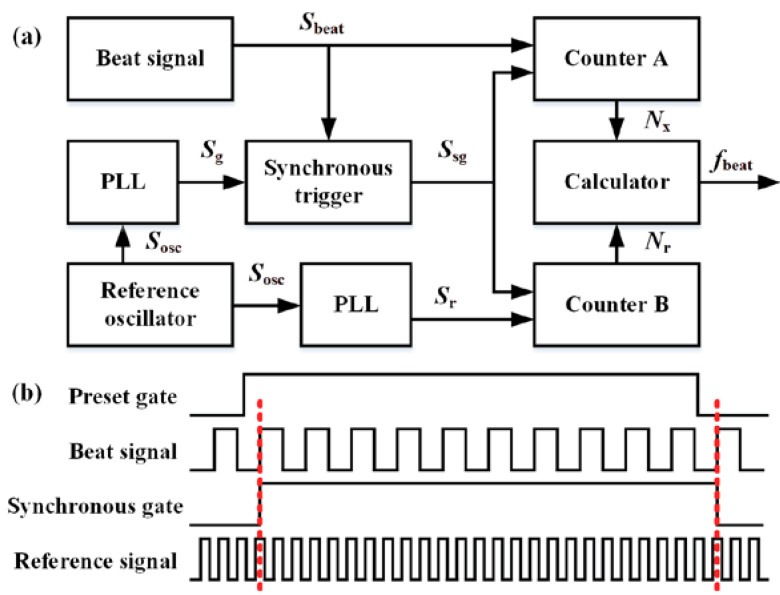
(**a**) Schematic of the synchronous multi-period frequency measurement method. (**b**) Timing sequences of the key signals during the measurememt. PLL: phase lock loop. *S*_beat_: beat signal. *S*_g_: preset gate signal. *S*_sg_: synchronous gate signal. *S*_osc_: oscillator signal. *S*_r_: reference signal. *f*_beat_: frequency of beat signal.

The schematic of the synchronous multi-period frequency measurement is shown in Figure 4a. The signal from a reference oscillator *S*_osc_ was converted by two phase lock loops (PLLs) to generate the preset gate signal *S*_g_ and reference signal *S*_r_, respectively. To eliminate the ±1 error from frequency counting of the beat signal, real gate signal *S*_sg_ was synchronized with the beat signal *S*_beat_. Gate signal *S*_sg_ triggered a pair of frequency counters for beat signal *S*_beat_ and reference signal *S*_r_. The corresponding counting numbers are presented as *N*_x_ and *N*_r_, respectively. Thus, the frequency of beat signal *f*_beat_ can be calculated as follows:

(7) $$f_{beat}=f_{r}N_{x}/N_{r},$$

where *f*_r_ is the frequency of the reference signal, which is determined by the oscillator frequency and the PLL index. 

As shown in Figure 4b, frequency counting of the reference signal may contain the ±1 error. Therefore, the relative accuracy of the measured beat signal frequency can be estimated as follows:

(8) $$\frac{df_{beat}}{f_{beat}}=\pm \frac{1}{T_{g}f_{r}},$$

where *T*_g_ is the time of the synchronous gate signal at high-level situations. Equation (8) suggests that the relative accuracy of the synchronous multi-period frequency measurement method is insensitive to the frequency of the beat signal. This property makes it suitable for measuring the beat signal with a large drift range.

## 4. Experimental Setup

**Figure 5:**
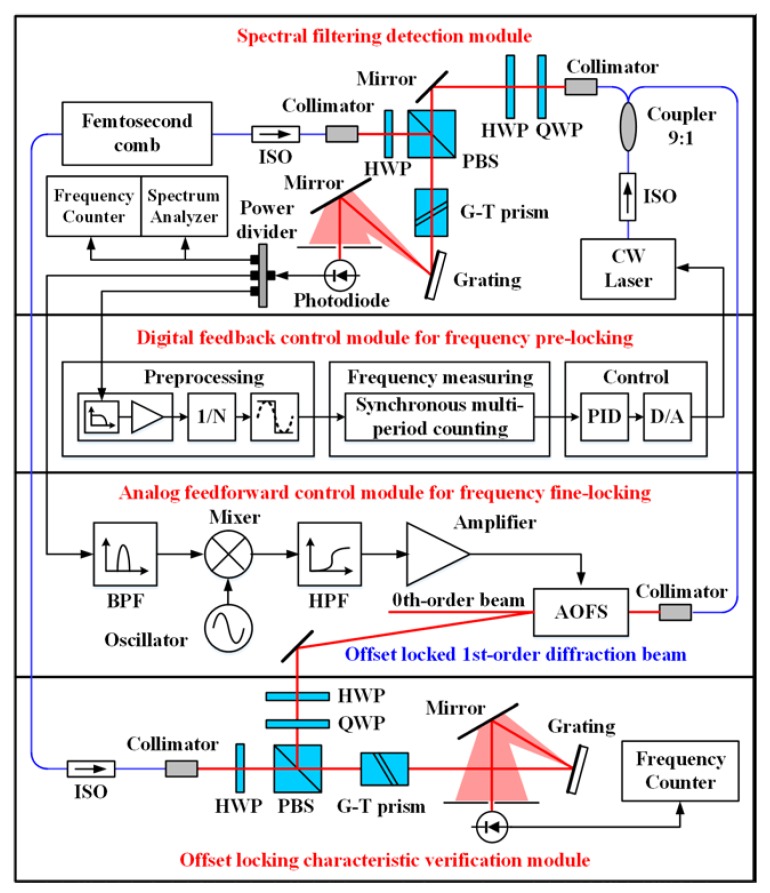
Schematic of the locking method with digital feedback and analog feedforward compound control. ISO: Isolator. PBS: Polarization beam splitter. HWP: Half-wave plate. QWP: Quarter-wave plate. G-T prism: Glan-Thompson prism. BPF: Band-pass filter. HPF: High-pass Filter. AOFS: Acousto-optic frequency shifter.

The experimental setup is illustrated in Figure 5. A 250 MHz Er-fiber femtosecond laser of 1550 nm was applied to provide the reference frequency comb with an original average power of 16 mW. The repetition rate and the carrier-envelope frequency of this frequency comb were both stabilized to the 10^−9^ level. The slave CW laser was a single frequency diode laser with compact butterfly package. In order to extract the beat signal between the CW laser and the reference comb with a high signal-to-noise ratio (SNR), the frequency comb was preselected by reducing the noise from the unneeded comb modes with a grating and a pinhole [19]. The CW laser and the reference comb were combined with a polarization beam splitter. Two half-wave plates were used to optimize the power of each laser individually and continuously. A Glan-Taylor prism was used as the high-efficiency polarizer. The combined beam was dispersed by a reflection grating. The CW laser spot of dispersive beams was aimed at the photodiode. The limited detection area of the photodiode acted as a pinhole to extract the desired beat signal from the massive inter-comb interference. With the help of this spatial filtering detection module, the SNR of the beat signal was highly improved up to 41 dB. A more detailed introduction of the spatial filtering detection module can be found in Section 5.1.

The frequency locking was achieved by combining a coarse digital feedback control and a fine analog feedforward control. For the digital feedback control module, a low-pass filter of 100 MHz cut-off frequency was applied in the signal preprocessing unit. The lower frequency beat signal could then be extracted. Since the frequency control coefficient of the CW laser was tested as ~1.02 GHz/V, a high-accuracy 16-bit DA convertor was applied in the system. 

In contrast, the feedforward control was realized for frequency fine-locking with a pure analog signal path. An AOFS was inserted into the beam of the CW laser to implement the feedforward control. After bandpass filtering, the beat signal was mixed with a local signal to enable the adjustment of the offset frequency. The mixed higher-frequency signal was amplified to drive the AOFS. Consequently, the frequency of the up-shifting first-order diffraction beam was compensated precisely.

In order to verify the frequency locking characteristic quantitively, another spectral filtering unit was established to detect the beat signal between the reference comb mode and the offset locked first-order diffraction beam. The basic structure is the same as the spectral filtering detection module. However, the monitoring device was changed to a frequency counter.

## 5. Experimental Results and Discussion

### 5.1. Detection of the Beat Signal with the Optimized Signal-To-Noise Ratio

The high-precision feedforward control relies on a beat signal with a high SNR. The major limitation of the beat signal SNR was attributed to the shot noise during weak interference signal detection, caused by the redundant comb modes. The spectral filtering detection module can reduce the number of redundant modes and improve the SNR of the beat signal significantly. According to the theoretical analysis in [19], the SNR of the beat signal with spectral filtering detection can be expressed as follows:

(9) $$SNR=\frac{\eta }{hvB_{\mathrm{PD}}}\frac{rP_{n}\left(1-r\right)P_{\mathrm{CW}}}{NrP_{n}+\left(1-r\right)P_{\mathrm{CW}}},$$

where *η* stands for the conversion efficiency of the photodetector, *hv* stands for the energy of a single photon, and *B*_PD_ is the bandwidth of the photodetector. *rP*_n_ and (1−*r*)*P*_CW_ are the detected power of the nth comb mode and the CW laser, respectively. The number of detected comb modes *N* can be calculated using the distance between the grating and pinhole *L* and the diameter of pinhole *d*:

(10) $$N\approx \frac{d}{L\Delta \lambda_{rep}\Delta \theta },$$

where Δ*λ*_rep_ and Δ*θ* are the spectral difference between adjacent comb modes and the angular dispersion of the grating. According to the 250 MHz repetition rate and 1550 nm center wavelength of the reference frequency comb, the spectral difference between adjacent comb modes was estimated as 2 pm. For the blazed grating with a groove spacing of 1/600 mm and a diffraction angle of 28°41′, the angular dispersion of first-order diffraction beam was calculated as 684 μrad/nm. The diameter of the pinhole was 0.5 mm. The distance between the grating and the photodetector was set to 200 mm. Therefore, the total number of detected comb modes *N* was estimated as 1827 in our setup. The InGaAs photodetector applied in our system was PDA10CF-EC from Thorlabs, whose conversion efficiency *η* and bandwidth are 1.04 A/W and 150 MHz, respectively. The detected power of the CW laser and the single comb mode were measured as 5.75 mW and 0.46 μW. According to Equation (9), the ideal SNR of the beat signal was estimated using the known parameters as 46.5 dB. 

**Figure 6:**
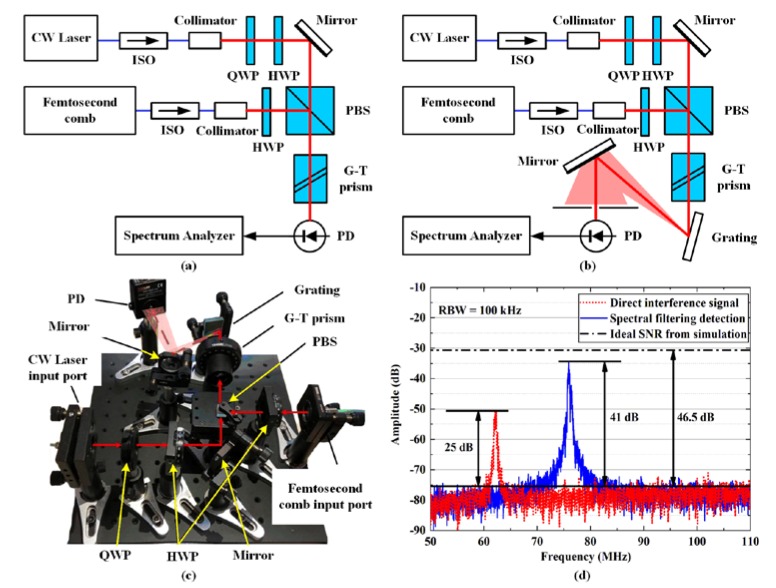
(**a**) The schematic of the direct beam combination detection module. (**b**) The schematic of the spectral filtering detection module. (**c**) The photo of the practical spectral filtering detection module. (**d**) The spectrum of beat signals from direct beam combination (red) and spectral filtering detection (blue). ISO: Isolator. PBS: Polarization beam splitter. HWP: Half-wave plate. QWP: Quarter-wave plate. G-T prism: Glan-Thompson prism. RBW: resolution bandwidth.

The beat signal between the CW laser and the reference comb was detected using a direct beam combination and a spectral filtering module for comparison. The schematic of direct combination and spectral filtering detection modules are shown in Figure 6a,b, respectively. The only difference was guiding the combined beam directly to the photodetector or to the grating first. Figure 6c is a photo of the practical spectral filtering module. The spectrum of beat signals from direct beam combination and spectral filtering detection are presented in Figure 6d with red dotted and blue solid lines, respectively. The SNR of the beat signal using the spectral filtering detection module was up to 41 dB, which is 16 dB higher than that of the direction combination method. 

It should be noticed that the SNR of beat signal is affected by the resolution bandwidth (RBW) of the spectrum detection [20]. In order to compare the SNRs under a unified standard, the RBW should be set to the same. Otherwise, the SNRs of different RBWs should be converted with the following equation for a fair comparison.

(11) $$SNR_{1}=SNR_{2}+10\mathrm{lg}RBW_{2}-10\mathrm{lg}RBW_{1}$$

In our experiments, the RBW was set as 100 kHz. With the same spectral filtering detection scheme and detection RBW, the beat signal SNRs between CW laser and frequency comb from some literatures are compared and shown in Table 1. The comparison result clearly shows that an optimal design of the detection system can further improve the achievable SNR, even with the same detection scheme. Moreover, the optimized SNR of the spectral filtering detection method was only 5.5 dB less than that of the theoretical simulation, which is shown in Figure 6d with a black dash-dotted line. We attribute the error between the simulated and experimental results mainly to the thermal noise of the photodetector, which was not considered in our analysis. Of course, an over estimation of the conversion efficiency *η* may also lead to the error.

### 5.2. Individual Performance of the Digital Feedback and Analog Feedforward Control Modules

**Figure 7:**
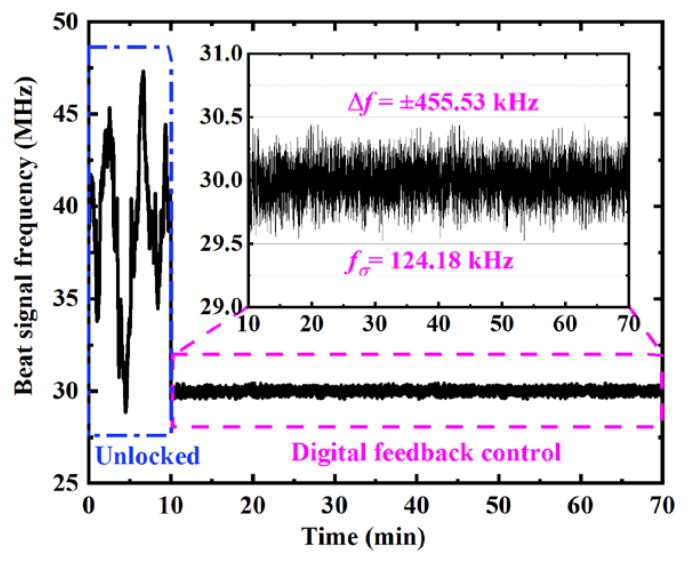
The frequency of beat signal between the CW laser and the reference frequency comb with a sample time of 0.1 s. In the first 10 min, the CW laser was unlocked. The corresponding experimental result was marked with a dash-dotted frame (blue). The digital feedback-controlled result was shown in the last 60 min and marked with dash frame (magenta). The insert is the local enlargement of the digital feedback result.

To verify the characteristic of the offset locking system comprehensively, the frequency pre-locking result of the digital feedback module was tested first. A frequency counter of Agilent 53230A with a gate time of 0.1 s was applied to evaluate its performance. First, the unlocked beat signal was extracted from the spectral filtering detection module and detected for 10 min. Then, we enabled the digital feedback control module for 60 min to test the pre-locking feature. The experimental results are displayed in Figure 7. During the 10 min unlocked stage, the drift range of the beat signal frequency was up to 20 MHz. When the digital feedback module was working, by contrast, the frequency of beat signal was locked to 30 MHz. The variation and the standard deviation of the beat frequency were ±455.53 kHz and 124.18 kHz, respectively. As the frequency control coefficient of the CW laser is approximately 1.02 GHz/V, the variation of beat frequency implies that the short-term stability of the control signal is better than ±0.46 mV. The digital feedback control module compensated the drift of the beat frequency and pre-locked it within the ±1.65 MHz range very well.

**Figure 8:**
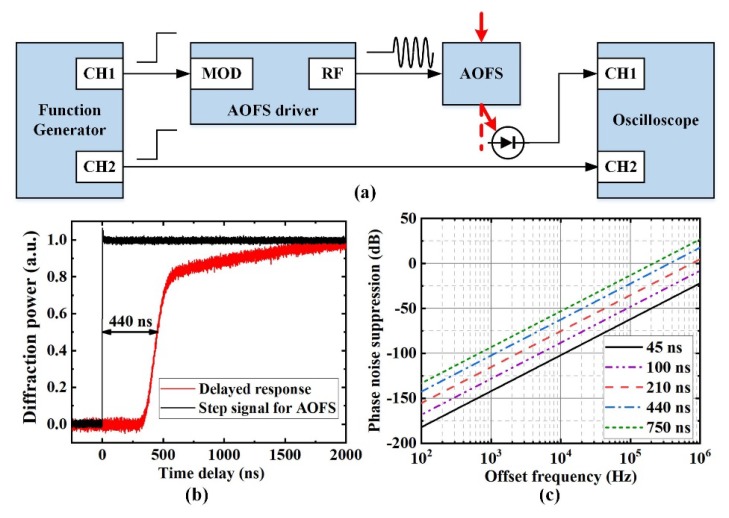
(**a**) Schematic of the time delay detection setup for AOFS. (**b**) Time response of the AOFS for a step signal. (**c**) The phase noise suppression property of the feedforward control with different offset frequency *v* and system time delay Δ*t*. CH1: channel 1; CH2: channel 2; MOD: modulation input port; RF: radio-frequency signal output port; AOFS: acoustic-optic frequency shifter.

As discussed in Section 2, the performance of the analog feedforward control module can be verified using the bandwidth and the phase noise suppression factor. The bandwidth of the feedforward control is determined by the feedforward time delay Δ*t*. Since Δ*t* is mainly caused by the AOFS module time delay, an experimental setup was realized based on the step response method (Figure 8a). Two step signals were provided by a dual-channel function generator synchronously. One step signal was transferred to the driver of the AOFS to enable the diffraction function of the AOFS. The increase in the power of the first-order diffraction beam was detected and compared with the other synchronous step signal using a dual-channel oscilloscope (Figure 8b). The time delay of the AOFS module was estimated as 440 ns. According to Equation (6), the control bandwidth can be estimated as 380 kHz. In order to further improve the control bandwidth, the feedforward delay time may be reduced from the following aspects. Firstly, the delay time from the signal cables can be minimized by a careful design of the feedforward control setup. Moreover, the transmission time of the acoustic wave should be reduced by aligning the CW laser beam as close as possible to the acoustic wave generator of the AOFS. With this method, the feedforward time delay and control bandwidth were improved to be 210 ns and 795 kHz, respectively [17]. However, this alignment optimization requires free-space AOFS with customized shell. 

At the same time, the factor of the phase noise suppression (2π*v*Δ*t*)^2^ was simulated (Figure 8c). The phase noise from the original CW laser dramatically decreased with the decrease in Δ*t*. For the system delay of 440 ns in our system, the phase noise suppression factor at 100 kHz was estimated as −24.5 dB. As the linewidth of our CW laser is approximately 100 kHz, the original phase noise from the CW laser can be significantly decreased.

### 5.3. The Verification of Offset Locking Characteristic

**Figure 9:**
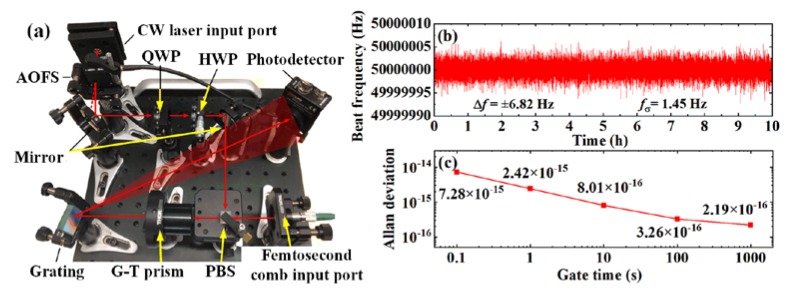
(**a**) Photo of the practical optical setup for feedforward control and offset-locking verification. (**b**) The beat frequency between the first-order diffraction CW laser and the reference frequency comb with a sample time of 0.1 s over a period of 10 h. (**c**) The Allan deviation of the offset-locked beat frequency.

The complete offset-locking system was realized with both the digital feedback and analog feedforward compound control modules. To eliminate the rapid drift of the unlocked beat frequency, the digital feedback control module was operating all along with the experiment. The analog feedforward module was manually enabled after the stabilization of digital pre-locking. As shown in Figure 9a, an extra spectral filtering detection module was established to verify the frequency stability of the offset-locking. The input laser beams were the first-order diffraction CW laser and the reference frequency comb. After the up-shifting of 80 MHz frequency with the AOFS, the beat frequency changed to 50 MHz. The frequency of the beat signal was measured with a sample time of 0.1 s over a period of 10 h. As shown in Figure 9b, the variation and the standard deviation of the beat frequency were ±6.82 Hz and 1.45 Hz, respectively. There was almost no drift in the experimental result, thus proving the long-term stability of the digital feedback and analog feedforward offset-locking system. The Allan deviations of the beat frequency are displayed in Figure 9c as 8.01 × 10^−16^ and 2.19 × 10^−16^ for a gate time of 10 s and 1000 s, respectively. 

The characteristic of the offset-locking setup was tested over 20 times in the past year. The maximum variation of the beat frequency was lower than ±6.97 Hz. Considering the center wavelength of 1550 nm, the relative offset-locking stability was lower than ±3.62 × 10^−14^. As most of the existing researches focus on the coherence transmission [10,12,13,14] or linewidth narrowing [16,17,18] property of the locking system, we only found a relative frequency stability of 3 × 10^−9^ in reference [11] for comparison. To improve the characteristic of the offset-locking system in the future, the delay time of the feedforward control module should be further minimized. The influence of the beat frequency SNR should be analyzed in detail.

## 6. Conclusions

We developed a simple and robust frequency locking system based on the digital feedback and analog feedforward compound control method. The basic principle and control range limitation of the conventional AOFS-based feedforward method was analyzed. The SNR of the interference signal between the slave CW laser and reference frequency comb was detected using a grating based spectral filtering unit and optimized up to 41 dB, which was only 5.5 dB less than the theoretical maximum. With a digital feedback loop, the beat frequency of the two lasers was monitored and pre-locked from over tens of MHz to less than 1 MHz. Hence, the control range of the compound system can be expanded by over an order of magnitude. By feeding the pre-locking beat frequency forward to an AOFS, the slave CW laser was ultrastable offset locked to the reference frequency comb for 10 h with a frequency standard deviation of 1.45 Hz. The Allan deviations of the beat frequency were 8.01 × 10^−16^ and 2.19 × 10^−16^ for gate times of 10 s and 1000 s, respectively. The maximum relative variation of the offset-locked beat frequency was better than ±3.62 × 10^−14^ in the long-term verification of one year. Our work provides a solution to generate ultrastable, highly reproducible, single-mode CW laser for a wide variety of applications, including precision interferometry and high-resolution spectroscopy. Considering the rapid development of the micro-resonator based optical frequency combs [29,30] and lasers [31], we believe the presented scheme can combine the advantages of these on-chip devices and promote a broader application in the field of integrated photonics.

## Figures and Tables

**Table 1:** Comparison of the SNRs between CW laser and comb with spectral filtering detection.

Reference	Reported SNR (dB)	Reported RBW (kHz)	Equivalent SNR (dB) for RBW = 100 kHz
Telle, 1999 [21]	16	100	16
Jones, 2001 [22]	35	2	18
Vogel, 2001 [23]	25	100	25
Adler, 2009 [24]	40	10	30
Newbury, 2007 [25]	47	3	31.8
Quinlan, 2011 [26]	30	300	34.8
Ruehl, 2011 [27]	38	100	38
Baumann, 2009 [28]	65	0.3	39.8
**This work**	**41**	**100**	**41**
